# Meiotic pairing and gene expression disturbance in germ cells from an infertile boar with a balanced reciprocal autosome-autosome translocation

**DOI:** 10.1007/s10577-016-9533-9

**Published:** 2016-08-02

**Authors:** Harmonie Barasc, Annabelle Congras, Nicolas Mary, Lidwine Trouilh, Valentine Marquet, Stéphane Ferchaud, Isabelle Raymond-Letron, Anne Calgaro, Anne-Marie Loustau-Dudez, Nathalie Mouney-Bonnet, Hervé Acloque, Alain Ducos, Alain Pinton

**Affiliations:** 1 GenPhySE, Université de Toulouse, INRA, INPT, ENVT, Toulouse, France; 2 LISBP, Université de Toulouse, CNRS, INRA, INSA, Toulouse, France; 3 GenESI Génétique, Expérimentation et Système Innovants, 17700 Saint-Pierre-d’Amilly, France; 4 STROMALab, Université de Toulouse, CNRS ERL 5311, EFS, ENVT, Inserm U1031, UPS, Toulouse, France

**Keywords:** Infertility, Meiosis, Reciprocal translocation, MSUC, Recombination, MSCI disturbance

## Abstract

Individuals carrying balanced constitutional reciprocal translocations generally have a normal phenotype, but often present reproductive disorders. The aim of our research was to analyze the meiotic process in an oligoasthenoteratospermic boar carrying an asymmetric reciprocal translocation involving chromosomes 1 and 14. Different multivalent structures (quadrivalent and trivalent plus univalent) were identified during chromosome pairing analysis. Some of these multivalents were characterized by the presence of unpaired autosomal segments with histone γH2AX accumulation sometimes associated with the XY body. Gene expression in spermatocytes was studied by RNA-DNA-FISH and microarray-based testis transcriptome analysis. Our results revealed a decrease in gene expression for chromosomes 1 and 14 and an up-regulated expression of X-chromosome genes for the translocated boar compared with normal individuals. We hypothesized that the observed meiotic arrest and reproductive failure in this boar might be due to silencing of crucial autosomal genes (MSUC) and disturbance of meiotic sex chromosome inactivation (MSCI). Further analysis revealed abnormal meiotic recombination (frequency and distribution) and the production of a high rate of unbalanced spermatozoa.

**Electronic supplementary material:**

The online version of this article (doi:10.1007/s10577-016-9533-9) contains supplementary material, which is available to authorized users.

## Introduction

Balanced constitutional reciprocal translocations are the most commonly identified structural chromosomal rearrangements in humans and pigs (frequencies of 0.1 and 0.4 %, respectively—Benet et al. 2005; Ducos et al. 2007). Although carriers are generally phenotypically normal, such rearrangements frequently lead to reproductive disorders. Indeed, the prevalence of reciprocal translocations is about 10 times higher in infertile men and some carriers present spermatogenesis disturbances leading to oligospermia or azoospermia, sometimes associated with teratosermia and/or asthenospermia (Dong et al. 2012; Mau-Holzmann 2005; Van Assche et al. 1996). Reciprocal translocations are also responsible for recurrent miscarriages and congenital defects in the offspring of carrier parents (Benet et al. 2005).

In reciprocal translocation heterozygotes, homologous regions of the normal and derivative chromosomes involved in the rearrangement pair during the prophase of the first meiotic division, due to the synaptonemal complex (SC), and form a particular structure called a multivalent (Oliver-Bonet et al. 2005; Villagomez and Pinton 2008). In some cases, chromosomal regions within the multivalent, especially around the breakpoints, remain unsynapsed which may trigger meiosis checkpoints leading to spermatogenesis arrest at the pachytene stage (MacQueen and Hochwagen 2011; Roeder and Bailis 2000). Several hypotheses have been proposed to explain such effects of pairing failure on gametogenesis. The first concerns altered transcription of the genes located on the unpaired segments. Indeed, studies in mice revealed transcriptional repression of unpaired regions by a specific mechanism called “meiotic silencing of unsynapsed chromatin” (MSUC) in individuals with partial or total spermatogenesis arrest (Turner et al. 2005). If some genes essential to the correct course of meiosis are located in these unsynapsed regions of the genome, MSUC may lead to the halting of meiotic division. Secondly, associations between the quadrivalent and the XY bivalent, which is transcriptionally silenced by a phenomenon known as meiotic sex chromosome inactivation (MSCI, Turner 2007), were also observed in individuals with altered semen parameters (azoospermic or oligospermic) (Oliver-Bonet et al. 2005; Sciurano et al. 2007, 2012). Such an association could result in partial reactivation of the XY body, leading to the expression of some genes located on the X chromosome (Lifschytz and Lindsley 1972) or spreading of XY body inactivation towards the autosomal segments attached to the XY body, without reactivation of this latter (Jaafar et al. 1993). Both mechanisms induce abnormal genetic expression, possibly responsible for gametogenesis failure. Both hypotheses were confirmed by Homolka et al. (2007) who simultaneously observed the reactivation of some X chromosome genes and the repression of autosomal genes located on unpaired segments, in a case of autosomal reciprocal translocation in mice.

Apart from the above-mentioned potential spermatogenesis failure, reciprocal translocations are systematically responsible for the production of genetically unbalanced gametes (Benet et al. 2005), which can subsequently be responsible for the reported miscarriages (embryonic mortality) or newborn defects. Indeed, chromosomes in the quadrivalent may segregate via different mechanisms (i.e., alternate, adjacent I, adjacent II, 3:1 and 4:0), resulting in the possible production of 18 different types of gametes. Only some types of segregation mechanisms produce genetically balanced gametes which contain either the two normal or the two derivative chromosomes (i.e., alternate segregation, or adjacent I segregation with a crossing-over (CO) in the interstitial regions of the quadrivalent—Benet et al. 2005; Sybenga 1975). The frequencies of the different modes of segregation can vary from one translocation to another and depend on the number and distribution of meiotic recombination sites on the quadrivalents, which, in turn, determine their configurations between prophase I and anaphase (chain or ring) (Faraut et al. 2000). Reciprocal translocations can also lead to disturbances in meiotic recombination. These disturbances may only concern the chromosomes involved in the rearrangement. A decreased number of mutL homolog 1 (MLH1) foci on the quadrivalent, as compared to the corresponding bivalents, has been reported for different translocations (Ferguson et al. 2008; Leng et al. 2009; Oliver-Bonet et al. 2005; Pigozzi et al. 2005a). Such disturbances may also be more general and affect the segregation of other chromosomes, leading to aneuploid gametes. On some occasions, the percentage of aneuploidy is inversely correlated to the ejaculated sperm concentration (Douet-Guilbert et al. 2005; Rives et al. 1999; Vegetti et al. 2000).

In the cases of autosome-autosome translocations in pigs, the semen parameters of carriers were generally normal, and SC analyses did not reveal any particular chromosome pairing behavior which could explain meiotic cell death (Villagomez and Pinton 2008). In fact, reciprocal chromosome translocations in the pig species tend to undergo heterosynapsis during the early pachytene stages, a germ cell mechanism avoiding apoptosis and subsequent meiotic arrest (Gabriel-Robez et al. 1988; Jaafar et al. 1992; Villagomez et al. 1993).

Here, we report for the first time in pigs a case of autosome-autosome translocation associated with oligoasthenoteratospermia (OAT) and characterized by the presence of a tiny derivative chromosome prone to disrupted meiotic pairing.

## Materials and methods

### Animals

The reciprocal translocation t(1;14) was initially identified during national systematic controls of young boars to be used in artificial insemination centers. The carrier individual (Landrace × Duroc crossbred boar) was phenotypically normal, but presented abnormal semen parameters: 19 × 10^6^ spz/mL (instead of 250 × 10^6^, on average, in normal individuals), with only 5 % of live spermatozoa with good motility and 78 and 66 % with acrosome and distal droplets abnormalities, respectively. Plasmatic levels of luteinizing hormone (LH), progesterone, testosterone, and androstenedione were within normal ranges (0.1 ng/mL, 1 nmol/L, 22 nmol/L, and 9 nmol/L, respectively). Five sows were inseminated with sperm from this boar, but no pregnancy could be obtained.

Testicular samples were collected by surgical hemi-castration. Pre-anesthesia (intramuscular injection of ketamine, 10 mg/kg; Virbac, Carros, France) was followed by inhalation anesthesia (isoflurane; Virbac, Carros, France). Post-operative follow-up was carried out in a recovery room adjacent to the operating facility. The animal was monitored until he recovered approximately 15 min after the operation. Castration was minimally invasive. Pain was relieved by an intramuscular injection of Finadyne (2 mg/50 kg; MSD Sante animale, Beaucouze, France) which completed the pre-anesthetic analgesia. The times of first getting up and first meal were monitored, and the healing process was controlled daily for 2 weeks.

### Cytogenetic and molecular characterization of breakpoints

Classical cytogenetic analysis (GTG banding) resulted in the identification of a highly asymmetrical reciprocal translocation involving the chromosomes *Sus Scrofa* chromosome 1 (SSC1) and 14 (SSC14).

The chromosomal breakpoints were finely localized by an array painting technique as described by Gribble et al. (2009). The two derivative chromosomes (1^14^ and 14^1^) (10 copies of each) were isolated from the GTG-banded metaphases by mechanical microdissection and their DNA amplified using the PicoPLEX WGA Kit (New Egland Biolabs, Ipswich, MA, USA). The specificity of this material was controlled on normal metaphases by fluorescence in situ hybridization (FISH) (Pinton et al. 2003).

The amplified DNA samples were differentially labeled with dUTP cyanine 3 (1^14^ chromosome) and dUTP cyanine 5 (14^1^ chromosome) and co-hybridized onto a pan-genomic custom chip of 4.2-M probes (Roche NimbleGen, Madison, WI, USA), designed from the porcine reference genome Sscrofa10.2. The microarray was then scanned, and the log_2_ ratios calculated from the intensities were plotted against chromosome position. The translocation breakpoints were defined as the positions where the log_2_ ratios changed from high to low ratios (or vice versa). The data were then confirmed by FISH using bacterial artificial chromosome (BAC) clones selected in the two breakpoint regions. The BACs CH242-298E5 (SSC1) and SBAB-234D5 (SSC14) were labeled with biotin using the BioPrime DNA Labeling System kit (Life Technologies, Carlsbad, CA, USA) and revealed by Alexa 594 conjugated to streptavidin (Molecular Probes, Eugene, OR, USA).

### Histopathological analysis of the testis sample

Testis samples were fixed in 10 % buffered formalin for 48 h before routine processing. Four-micrometer-thick paraffin sections were stained with hematoxylin and eosin.

Anti-caspase-3a immunohistochemistry was performed as already described (Cheat et al. 2015) with an active caspase-3 antibody at 1:300 dilution (R&D Systems, Minneapolis, MN, USA).

Briefly, 4-μm paraffin-embedded sections from a testis were dewaxed in toluene and rehydrated first in an acetone bath then in deionized water. Antigen retrieval was carried out in 10 mM citrate buffer at pH 6.0 for 30 min in a water bath at 95 °C. The cooled sections were then incubated in Dako peroxidase blocking solution (Dako, Glostrup, Denmark) to quench any endogenous peroxidase activity. Non-specific binding was blocked by incubation in normal goat serum at 1:10 dilution (Dako, Glostrup, Denmark) for 20 min at room temperature (RT). The primary antibody was anti-active caspase-3 (dilution 1:300) (R&D Systems, Minneapolis, MN, USA). Sections were incubated with primary antibodies for 50 min at RT. Bound primary antibodies were detected with EnVision™ + Horseradish Peroxidase (HRP) Systems (Dako, Glostrup, Denmark) for 30 min at RT. Peroxidase activity was revealed by 3,3′-diaminobenzidine tetrahydrochloride substrate (Dako, Glostrup, Denmark). Finally, the sections were counterstained with Harris hematoxylin, dehydrated, and coverslipped.

### Analysis of meiotic pairing

#### Immunocytology

Meiotic cells were prepared as described by Pinton et al. (2008) with some modifications. Detection of the synaptonemal complex proteins 3 (SCP3) and 1 (SCP1) and centromeres was carried out before immunostaining the γH2AX protein.

The meiotic proteins were immunolocalized using antibodies at 1:100 dilution in PBT (1× phosphate-buffered saline (PBS), 0.15 % bovine serum albumin (BSA), and 0.1 % Tween 20) as follows. First, the SCP1 and centromeres were detected using the following primary antibodies: rabbit anti-SCP1 (Abcan, Cambridge, UK) and human anti-centromere (Antibodies Incorporated, Davis, CA, USA). Secondary antibodies consisted of DyLight 488 conjugated goat anti-rabbit (KPL, Gaithersburg, MD, USA) and 1-amino-4-methylcoumarin-3-acetic acid (AMCA) conjugated donkey anti-human (Jackson ImmunoResearch Laboratories, Grove, PA, USA). Secondly, SCP3 was detected using rabbit anti-SCP3 (Abcam, Cambridge, UK) and then revealed with secondary antibody Alexa 594 conjugated donkey anti-rabbit (Molecular Probes, Eugene, OR, USA). Spermatocytes were captured using a Zeiss Imager Z2 microscope with CytoVision imaging system (Leica Microsystemes, Nanterre, France). Finally, the γH2AX protein was detected using mouse anti-γH2AX (Abcam, Cambridge, UK) and Alexa 488 conjugated goat anti-mouse (Molecular Probes, Eugene, OR, USA) antibodies.

#### Fluorescence in situ hybridization

After SC analysis, the same cells were subjected to FISH with BAC clones (SBAB-428G6, SBAB-413G8, SBAB-498D8 from the National Institute for Agricultural Research (INRA) BAC library) (Rogel-Gaillard et al. 1999): one was located in the telomeric region of the SSC1 p arm (labeled with biotin), and two were located on SSC14, one in the telomeric region (labeled with digoxigenin) and the other in the centromeric region (containing porcine endogenous retrovirus (PERV) sequences, differentially labeled with biotin and digoxigenin). These BAC clones were labeled using the BioPrime DNA Labeling System kit (Life Technologies, Carlsbad, CA, USA), and revealed by Alexa 594 conjugated to streptavidin (Molecular Probes, Eugene, OR, USA) and fluorescein isothiocyanate (FITC) conjugated mouse anti-digoxygenin antibodies (Sigma-Aldrich, Saint Louis, MO, USA). FISH signals from the same cells for which SCs had previously been analyzed were captured and evaluated using an Imager Z2 microscope with CytoVision imaging system (Leica Microsystemes, Nanterre, France).

### Recombination analysis

Analysis of meiotic recombination was carried out as described by (Mary et al. 2014). The quadrivalent was identified using the BAC clones previously mentioned in the “Fluorescence in situ hybridization” section. The images were analyzed using MicroMeasure 3.3 software (Reeves 2001) to determine the length of the SCs, as well as the positions of the centromeres and recombination sites (MLH1 foci).

### RNA-DNA FISH and fluorescence immunostaining of the γH2AX protein

PERV RNA-DNA FISH, coupled with immunolocalization of the γH2AX protein, was performed as described by Barasc et al. (2012). RNA integrity was preserved by carrying out the PERV RNA-FISH experiment before the immunostaining and PERV DNA-FISH.

### Microarrays for gene expression analysis

Total RNAs were extracted from testis samples from three fertile control boars with normal semen parameters (Supplementary Table 1) and from the t(1;14) boar using the method described in Congras et al. (2014). Three independent extractions from three samples were performed for each animal. RNA quantity and quality were measured by NanoDrop dosage and Bioanalyzer analysis (Agilent Technologies, Santa Clara, CA, USA). Samples were then labeled with cyanine 3 fluorochrome and converted into complementary DNA (cDNA) before hybridization on the 60K customized porcine microarray from Agilent (Congras et al. 2016). Only spots of sufficient intensity and quality for at least two out of three technical replicates were retained. Intensity values were log-transformed, normalized by quantile normalization method, then subjected to differential analysis using the Limma package (Bioconductor). The selected threshold parameters were 0.01 for the maximal adjusted *p* value (Benjamini and Hochberg 1995) and 2 for the minimal log-fold change (lfc).

Probes were mapped along the chromosomes by aligning all the microarray probe sequences on the pig reference genome (SGSC Sscrofa10.2/susScr3) using Blat software (Kent 2002) and then plotting the differentially expressed (DE) probes along the chromosome coordinates together with their Log_2_ ratio (microarray GEO accession codes: GSE80693)

### Analysis of gene expression in the testis by real-time PCR

Total RNAs used for the microarray study were converted to cDNA with Superscript II Reverse Transcriptase (Life Technologies, Carlsbad, CA, USA). Expression analysis of six genes located on the X chromosome and up-regulated in the microarray analysis was carried out by real-time qPCR using the primers listed in Supplementary Table 2 and the Mx3005P Real-Time PCR System from Agilent Technologies. Results were analyzed by the 2ΔΔCt method and statistical *t* test. RPL4 was used as the reference gene for relative quantification (Congras et al. 2016).

### Sperm FISH analyses

Sperm was prepared as reported by Bonnet-Garnier et al. (2009)

#### Meiotic segregation profiles

Sperm FISH analyses were carried out using the above-mentioned BAC clones as described in the Fluorescence in situ hybridization” section. The signals from 1582 spermatozoa heads were analyzed.

#### Interchromosomal effects

Painting probes were used for chromosomes SSCX and SSCY and for the autosomal controls SSC13 and SSC18. These probes were produced by degenerate oligonucleotide-primed polymerase chain reaction from flow sorted (SSC13, SSC18, and SSCY) or microdissected (SSCX) chromosomes. The SSCX probe was labeled with biotin, the SSCY probe with biotin and digoxigenin, and chromosomes 13 and 18 with digoxigenin. Biotin-labeled probes were revealed in red using Alexa 594 conjugated to streptavidin and amplified with rabbit anti-streptavidin coupled to Texas Red (Abcam, Cambridge, UK) and Alexa 594 conjugated donkey anti-rabbit antibodies. Digoxigenin-labeled probes were revealed in green by FITC conjugated mouse anti-digoxigenin and amplified with Alexa 488 conjugated goat anti-mouse and donkey anti-goat coupled to Alexa 488 (Molecular Probes, Eugene, OR, USA) antibodies.

Slides were analyzed under a Zeiss microscope fitted with a triple bandpass filter. More than 10,000 sperm nuclei were scored for each experiment. Only spermatozoa with signals of equivalent intensity and separated by a distance at least the size of one signal were counted.

### Statistical analysis (R software, Ihaka and Gentleman 1996)

Differences in the relative SC lengths in SSC1 and SSC14 between the translocated boar and controls were examined by applying the Wilcoxon test. The same test was used to compare the number of MLH1 foci (per cell, as well as on chromosomes 1 and 14) between the translocated boar and controls. The MLH1 distributions on SSC1 and SSC14 were compared using the Kolmogorov-Smirnov test. For the sperm FISH results, a classical 2 × 2 chi-square test with Yates continuity correction was used to compare the proportions of each sperm category in the t(1;14) boar and a control boar. *P* values <0.05 were considered statistically significant.

## Results

### Molecular characterization of the breakpoints

**Fig. 1:**
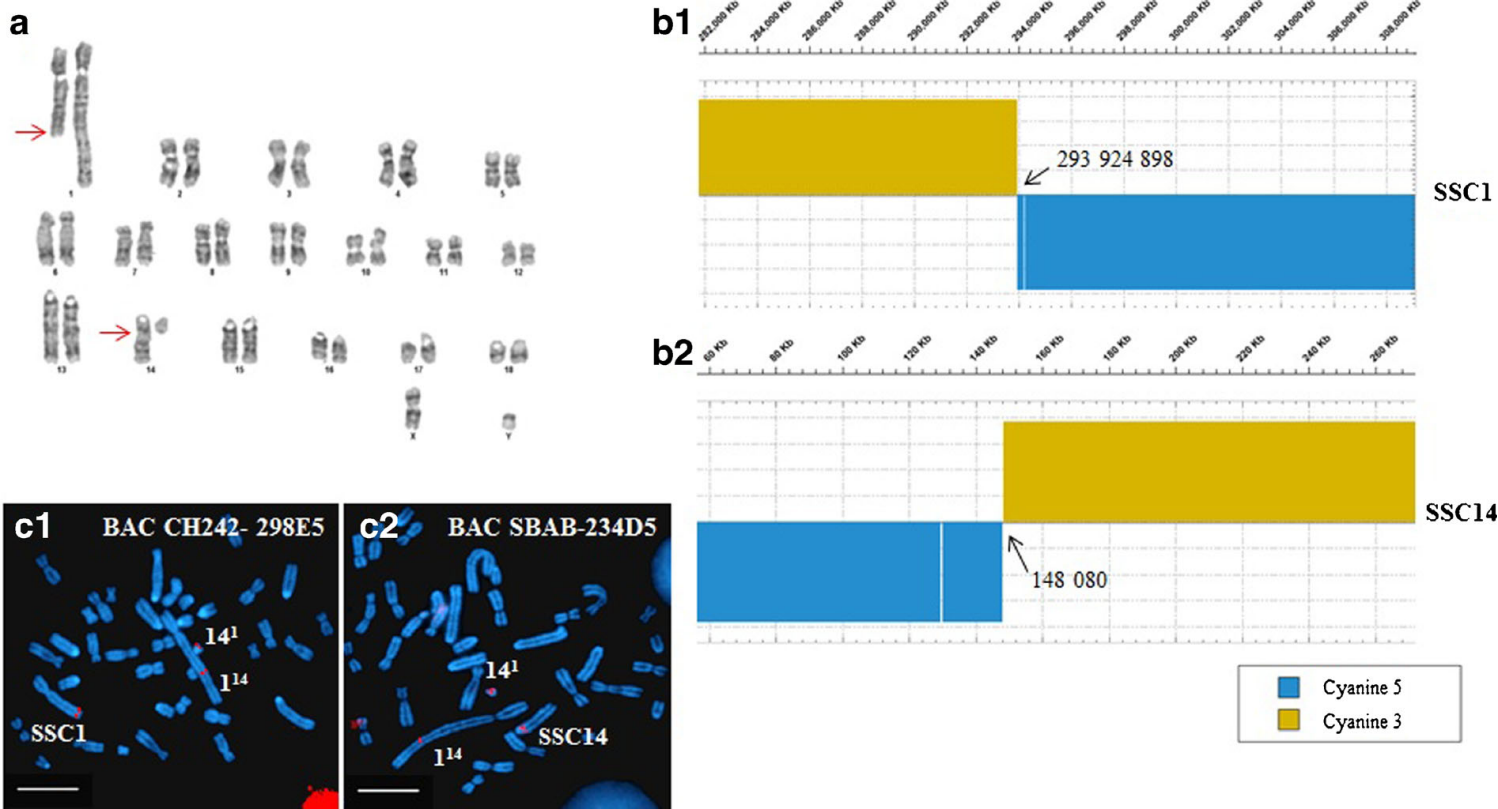
Characterization of the chromosomal rearrangement. **a** GTG-banded karyotype of the boar carrying a t(1;14)(q2.11;q1.2) translocation. *Arrows* indicate the locations of the breakpoints on the derivative chromosomes. **b** Array painting of derivative chromosomes 1 and 14 on a custom 4.2-M whole genome array designed from the porcine sequence Sscrofa10.2. Transitions in the hybridization profiles indicate the chromosome breakpoints: positions 293 924 898 (SSC1) (B1) and 148 080 (SSC14) (B2). **c** Confirmation of the array painting results by FISH of BAC clones overlapping the breakpoints: clone CH242-298E5 on SSC1 (BAC position: start 293811882–end 293992669) (*C1*) and BAC SBAB-234D5 on SSC14 (BAC position: start 134867–end 279323) (*C2*). These array painting results were confirmed by splitting of the BAC hybridization signals on the derivative chromosomes. *Scale bars* are all equal to 10 μm

Analysis of the GTG-banded karyotypes revealed a balanced reciprocal translocation between the q arm of one SSC1 chromosome and the q arm of one SSC14 chromosome (Fig. 1a). The breakpoint on SSC1 was very distal (q2.11 band), whereas the breakpoint on the acrocentric chromosome was located close to the centromere (q1.2 band). The array painting experiment accurately located the breakpoints at positions 293 924 898 (SSC1) and 148 080 (SSC14) (Fig. 1b) of the pig sequence assembly (Sscrofa10.2). These array painting results were confirmed by selecting BAC clones in the regions overlapping the breakpoints on both chromosomes and hybridizing them on to metaphases of the (1;14) boar (Fig. 1c). The hybridization signals for the two BAC probes were indeed observed to split on the two derivative chromosomes, thereby confirming the breakpoint positions.

### Histopathology

**Fig. 2:**
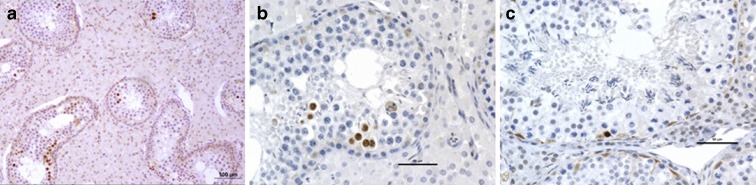
Histopathological analysis of testicular tissue. Anti-caspase-3 immunohistochemistry on paraffin sections from t(1;14) (**a**, **b**) and control (**c**) testis samples. Clusters of positive cells (dark brown staining of the nuclei) in moderately atrophic seminiferous tubules. (**a** ×100 magnification. **b**, **c** ×400 magnification) (anti-caspase-3a antibody). *Scale bars* are all equal to 100 μm and 50 μm respectively

Histopathological evaluation of the testis revealed diffuse moderate seminiferous atrophy associated with diffuse severe interstitial cell hyperplasia. Seminiferous tubules showed altered spermatogenesis with few spermatozoa produced in accordance with the oligospermia detected during sperm analysis (Supplementary Fig. 1). In addition, a significant increase in the number of apoptotic cells, identified as spermatocytes due to their nuclear size and structure (Barasc et al. 2012; Koykul et al. 2000; Pinton et al. 2008), was demonstrated by anti-caspase-3a immunohistochemistry (Fig. 2). Apoptotic cells were observed as small multifocal clusters of contiguous positive cells (probably due to the fact that they undergo apoptosis at the same developmental stage of meiotic prophase), often in the middle or luminal portion of the seminiferous epithelium (spermatocyte stage). Binucleated positive cells or both daughter cells positive were also regularly observed, showing that apoptosis probably occurred in cells derived from the same spermatogonia lineage with impaired meiosis (Fig. 2).

### Meiotic pairing

**Fig. 3:**
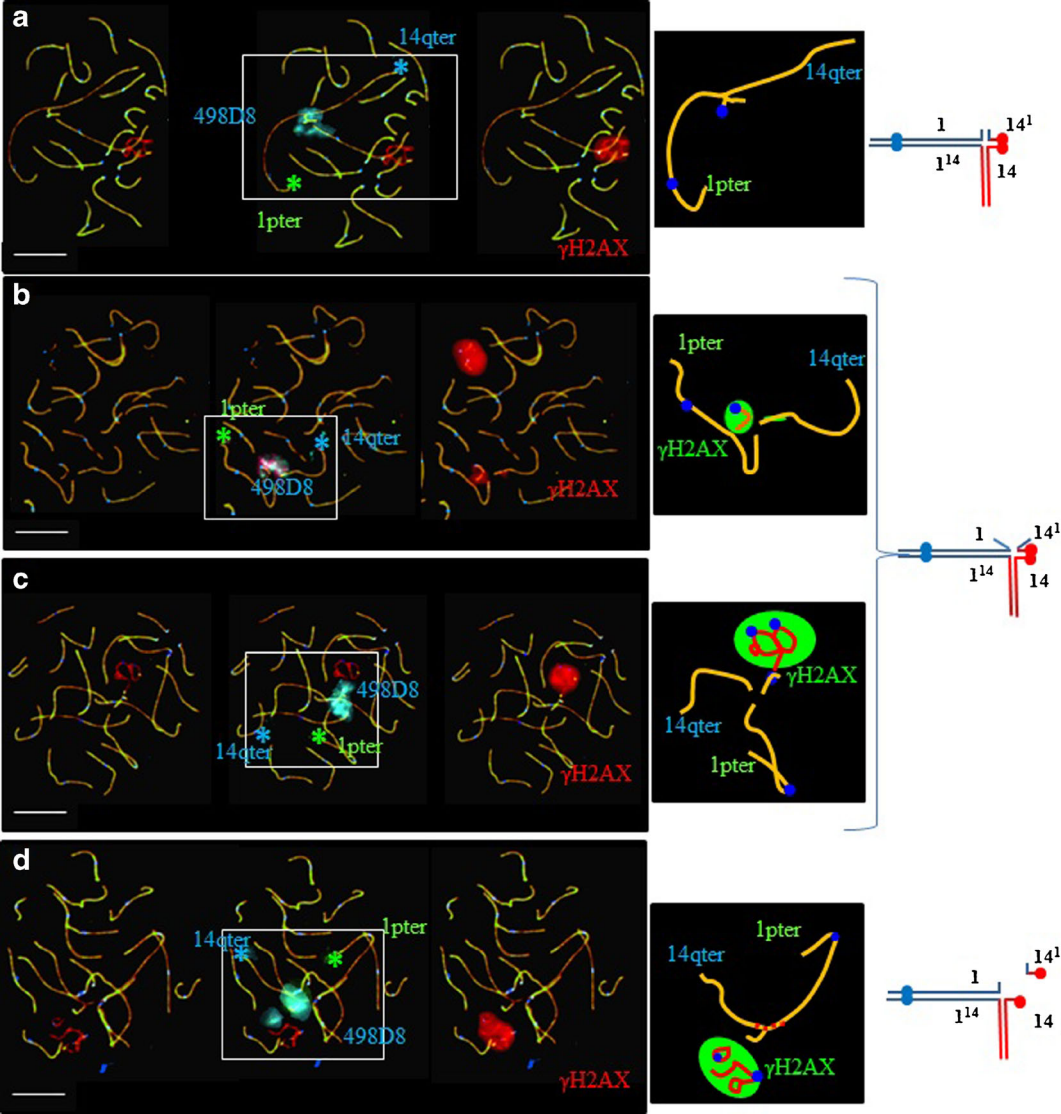
Analysis of meiotic pairing in pachytene spermatocytes using FISH and immunostaining of the synaptonenal complex proteins SCP1 (*green*) and SCP3 (*red*), as well as γH2AX (*red*) and the centromeres (*blue*). **a**–**c** Spermatocytes with a quadrivalent configuration. **a** Complete synapsis, no γH2AX signal on the quadrivalent, and no association with the XY body. **b** Unsynapsed segment with γH2AX signal on the derivative 14 chromosome but no association with the XY body. **c** Colocalization of the univalent (derivative 14 chromosome) with the XY body in a γH2AX-positive region. **d** Spermatocyte with a “trivalent plus univalent” configuration. The univalent is characterized by an absence of SCP1 signal (*red* synaptonemal complex instead of *orange*) and is included in a γH2AX-positive region with the XY body. *Scale bars* are all equal to 10 μm

SC analysis of 284 pachytene nuclei revealed two pairing configurations: formation of a quadrivalent in 70 % of the cells (Fig. 3a–c) and a “trivalent plus univalent” configuration in 30 % of the others (Fig. 3d). The univalents always corresponded to the small 14^1^ chromosome. Fifty-two percent of the 284 cells showed meiotic pairing abnormalities (γH2AX-positive unpaired autosomal regions associated or not with the XY body) (Fig. 3b–d).

### Recombination analysis

#### Overall recombination rate per cell

**Fig. 4:**
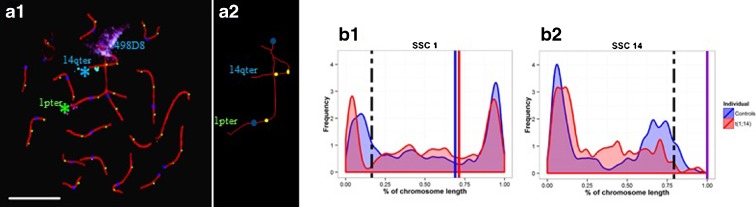
Analysis of meiotic recombination in the t(1;14)(q2.11;q1.2) boar. **a** Immunostaining of pachytene spermatocytes. Pachytene spermatocyte after immunostaining of centromeres in *blue*, MLH1 signals in *green*, SCs in *red*, and FISH (*A1*). Schematic representation of the quadrivalent observed in *A1* (*A2*). *Scale bar* represents 10 μm. Distributions of MLH1 foci on chromosomes 1 and 14 (*B1*, *B2*). Comparison between rearranged chromosomes and controls (*red curve*: t(1;14); *blue curve*: control boars); *red bar*: centromere position on SSC1 for translocated boar; *blue bar*: centromere position on SSC1 for control boars; *purple bar*: centromere position on SSC14 for all individuals; *dotted bar*: position of the breakpoints. The *x-axis* represents the percent of SC length, and the *y-axis* indicates the frequency of MLH1 signals

Autosomal MLH1 signals, markers of CO events during meiosis I, were counted in 80 pachytene spermatocytes from the boar carrying t(1;14)(q2.11;q1.2) (Fig. 4a). The results were compared with those obtained for a pool of three normal boars (two Large White and one Meishan) in the study by Mary et al. 2016 (Table 1). The average number of recombination events per cell was 33.42 in the t(1;14) carrier (range 25–42), i.e., significantly higher than the value of 31.86 (range 21–40) obtained for the control boars (*p* < 0.05).

**Table 1:** Average number of MLH1 foci per cell for chromosomes 1 and 14

Individuals	No. of cells analyzed	Average no. of recombination foci per cell (without sex chromosomes) and SE	Range	Reference
Controls	264	31.86 ± 0.18	21; 40	Mary et al. (2016)
t(1;14)(q2.11;q1.2)	80	33.42 ± 0.39*	25; 42	Present study

Wilcoxon test

*p < 0.05

#### Recombination rates on the chromosomes involved in the translocation

Recombination was only studied in the spermatocytes with a quadrivalent configuration. The relative lengths of the SCs, expressed as a percentage of the total length of all the SCs within a cell, and the number of recombination events (MLH1 foci per chromosome) were measured for chromosomes 1 and 14 (part of the chromatin corresponding to SSC1 and SSC14 in the quadrivalent of the boar carrying the translocation) and compared to the values obtained for normal boars (Table 2). Both the relative SC length and the number of MLH1 foci were significantly higher in the boar carrying the translocation than in the controls for chromosome 1 (*p* < 0.05). In contrast, no significant difference was observed for chromosome 14.

**Table 2:** Comparisons of the relative lengths of SCs and of the number of recombination foci for chromosomes 1 and 14, between the translocated boar and a control

Individuals	No. of cells analyzed	Chromosome	Relative length of SCs in % (1 SD)	Number of recombination foci (ISE)	Reference
Controls	264	1	10.64 10.96	2.74 10.04	Mary et al. (2016)
		14	8.15 11.28	1.94 10.03	
t(1;14)(q2.11;q1.2)	80	1	11.6511.39*	3.1210.09*	Present study
		14	8.15 11.24	1.91 10.07	

Wilcoxon test

*p < 0.05

#### Distributions of the MLH1 foci

The distributions of MLH1 foci observed along chromosomes 1 and 14 in spermatocytes from the boar carrying the translocation are presented in Fig. 4b. These distributions are clearly different (*p* < 0.05) from those obtained in the normal boars. The most important modifications were observed in the breakpoint regions, where a decrease in the number of MLH1 signals was noted.

### Analysis of transcriptional activity in the spermatocytes

**Fig. 5:**
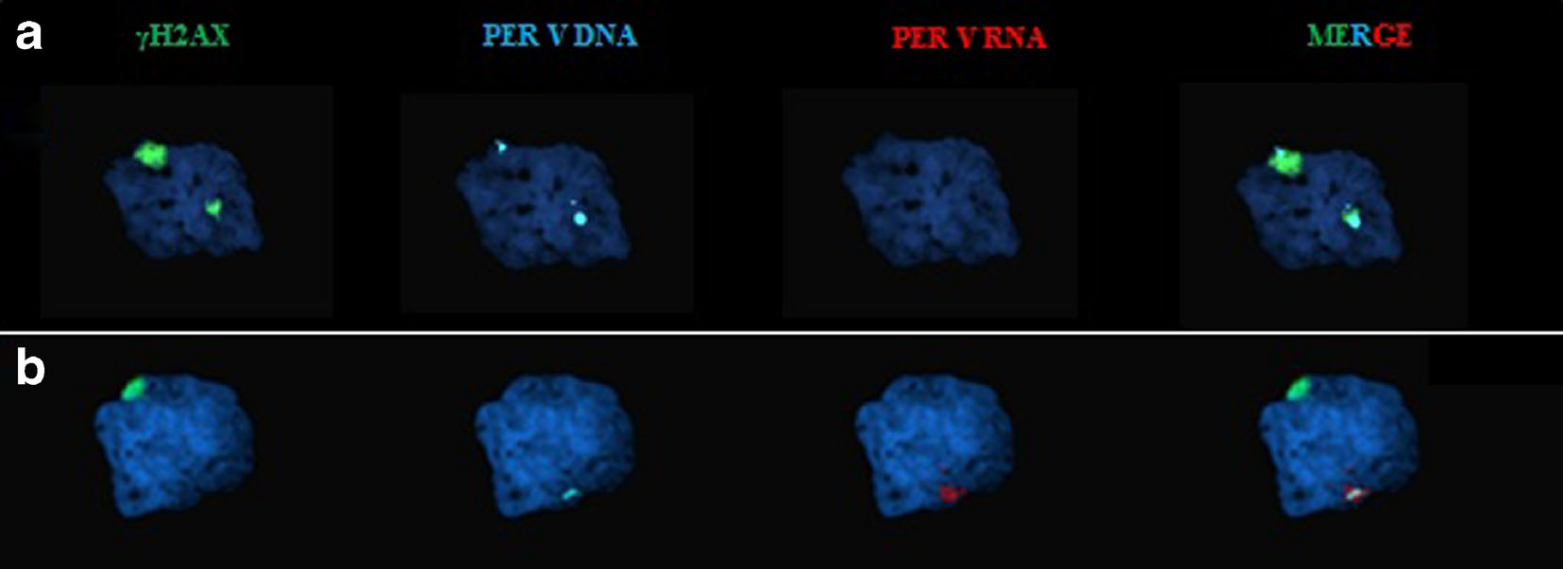
Analysis of PERV expression by sequential RNA- and DNA-FISH. **a** Spermatocyte showing a “trivalent plus univalent” configuration with two DNA PERV signals located in down-regulated γH2AX regions (no RNA signals). One of the PERV signals is co-localized with the large γH2AX region (probable XY body). **b** Spermatocyte showing a transcription of the PERV gene, located outside the γH2AX region. *Scale bars* are all equal to 10 μm

Spermatocytes from the translocated boar were analyzed by RNA-DNA FISH to detect PERV gene expression. Eighteen of the 35 spermatocytes analyzed showed co-localization of the PERV-DNA signal and γH2AX protein. No PERV-RNA signal was observed in these cells (Fig. 5a). The PERV-DNA signals in the 17 remaining cells were located outside the γH2AX-positive regions. In these cells, the PERV gene was expressed (co-localization of the DNA and RNA signals; Fig. 5b).

**Fig. 6:**
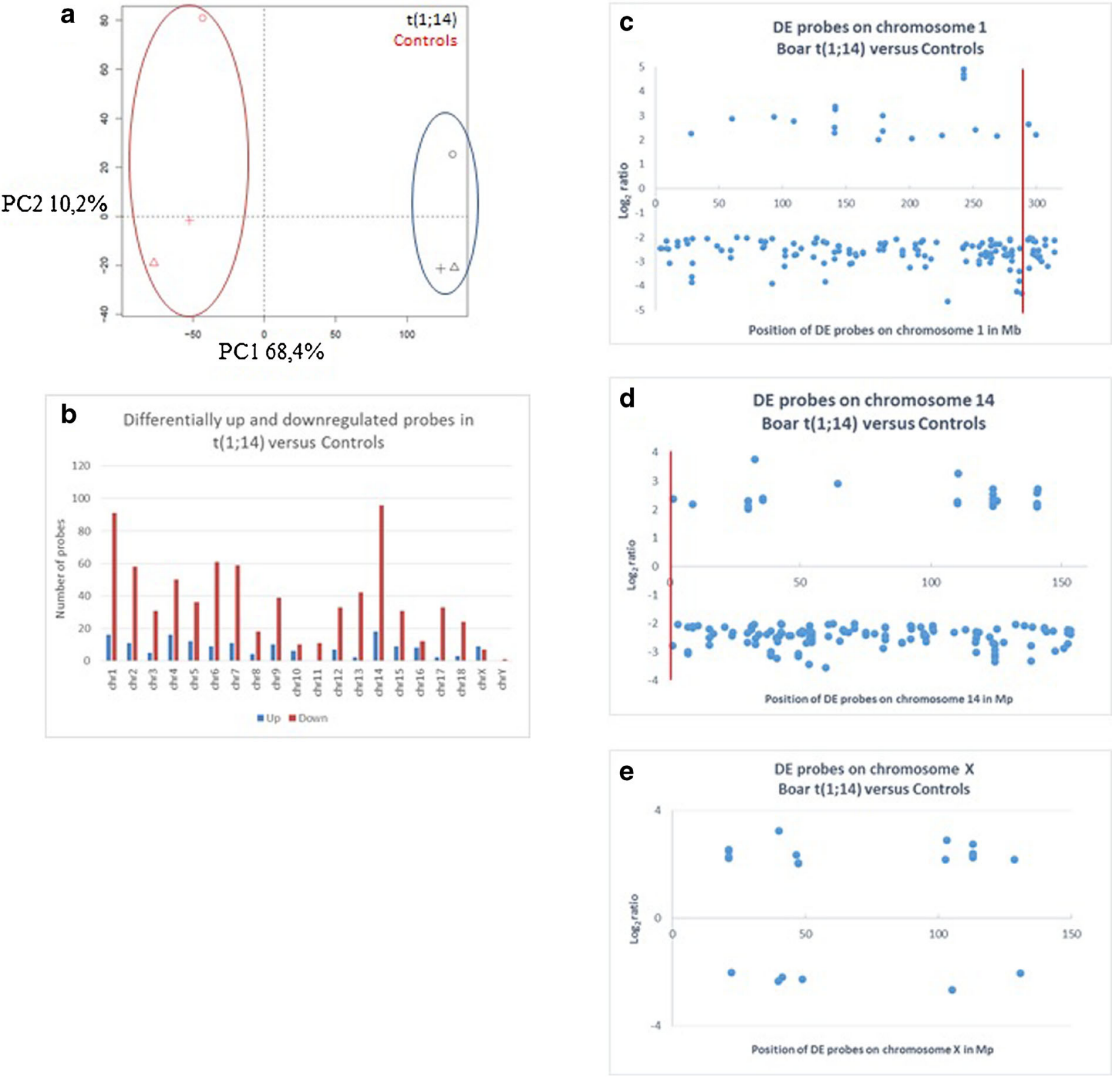
Gene expression profile from fertile and t(1;14) boar testis samples. **a** Principal component analysis of microarray transcriptomic data from testis RNA extracted from controls (*red*) and t(1;14) (*black*) boars. Gene expression profile from t(1;14) segregates differently from the control group and is reflected on axis 1 that explains 68.4 % of observed variance. The variance represented on axis 2 (10.2 % of variance) is mostly explained by the heterogeneity between experimental replicates (*circle*, *triangle*, and *cross*). **b** Number of differentially up-regulated (*blue bars*) and down-regulated (*red bars*) probes per chromosome in t(1;14) versus controls. Except for the X chromosome, down-regulated probes are always more abundant than up-regulated ones. **c**–**e** Mapping of differentially expressed probes (*blue dots*) on chromosome 1, chromosome 14, and chromosome X in t(1;14) versus controls. The *y-axis* corresponds to the log2 ratio where negative values correspond to down-regulated probes and positive values to up-regulated probes. Only probes with log2 values over 2 or under −2 were kept. The breakpoint is highlighted by a *vertical red line*

We also measured the effect of t(1;14) on gene expression in the testis using an Agilent 60K customized porcine microarray. This enabled us to compare the transcriptome of the t(1;14) boar with the transcriptome obtained from a pool of control boars with normal semen parameters. Principal component analysis (PCA) highlighted the differences between the two groups, with all biological replicates clustering together (Fig. 6a). Interestingly, the first axis (68.4 % of the variance) was explained by the variation between t(1;14) and control boars, while the second axis (10.2 % of the variance) was explained by the heterogeneity between experimental replicates. We then compared probe intensity between t(1;14) and controls. We observed 2278 differential probes for t(1;14). Within the differentially expressed probes, 1707 (75 %) corresponded to a decrease in expression while 571 (25 %) corresponded to an increase in expression. We then mapped the differential probes on the porcine chromosomes and observed that the two chromosomes with the largest number of differential probes were chromosome 1 and chromosome 14, mostly for down-regulated probes (Fig. 6b). The X chromosome was the only one with more up-regulated than down-regulated probes (Fig. 6b). Nine genes on the X chromosome, identified through the microarray study, were up-regulated (LAMP2, TMEM47, SAT1, RGN, L1CAM, ZIC3, TSC22D3, PLP2, ARAF1). Real-time PCR was performed on six of them (ARAF1, LAMP2, RGN, SAT1, TSC22D3 and ZIC3). All six tested genes were more expressed in the testis from t(1;14), and the increase in ARAF1, LAMP2, RGN, SAT1, and TSC22D3 was significant and more than twofold (or higher) in the testis from the t(1;14) boar than in control boars (Supplementary Fig. 2).

We then determined the precise locations of the differential probes on chromosome 1, chromosome 14, and chromosome X. We first used array CGH to localize the DNA breakpoints at positions 293 924 898 on chromosome 1 and 148 080 on chromosome 14 (red lines on Fig. 6c, d). We found that the density of differential probes around the breakpoint was increased on chromosome 1 but not on chromosome 14 (Fig. 6c, d). Similarly, the probes were homogeneously distributed on chromosome X, except for a big gap of 50 Mb around the centromeric region (Fig. 6e).

### Analysis of meiotic segregation profiles

The meiotic segregation profiles were studied using FISH of BAC clones on decondensed sperm heads (sperm FISH). These meiotic products originated from two different multivalent structures (quadrivalent or trivalent plus univalent configurations) in the presence (or not) of interstitial recombination. It was therefore impossible to determine the segregation mode at the origin of the different gametes unequivocally. We therefore employed the term “-like” for all the segregation products to indicate that their exact origin (segregation mode) could not be determined. The data are presented in Table 3.

**Table 3:** Segregation patterns for the boar carrying the (1;14) translocation

Segregation	Alternate		Adjacent I		Adjacent II		3:1							
Chromosomal constitution	1/14	1 ^14^ /14 ^1^	1/14 ^1^	1 ^14^ /14	1 ^14^ /1	14 ^1^ /14	1 ^14^	1/14/14 ^1^	1 ^14^ /14/14 ^1^	1	1 ^14^ /1/14 ^1^	14	1 ^14^ /1/14	14 ^1^
Number of gametes investigated	430		366	2	13	80	50	12	3	48	0	375	4	199
% By combinations	27		23		6		44							

The most frequently observed segregation products were “3:1-like” (44 %), followed by “alternate-like” (27 %), “adjacent I-like” (23 %), and “adjacent II-like” (6 %). The overall proportions of balanced and unbalanced spermatozoa were 27 and 73 %, respectively. In the “2:2-like” segregation products, a strong disequilibrium was observed between the proportions of reciprocal products: 99 %/1 % for the adjacent I segregation (instead of the theoretical 50 %/50 % distribution) and 86 %/14 % for the adjacent II segregation. Strong disequilibria were also noted for the 3:1-like segregation products. Two types of gametes in that category were highly prominent: sperm containing SSC14 only (54 % of all 3:1-like segregation products) and sperm containing the derivative chromosome 14 only (29 % of all 3:1-like segregation products).

### Analysis of interchromosomal effects

Aneuploidy rates for the SSC13, SSC18, SSCX, and SSCY chromosomes were estimated in the boar carrying the (1;14) translocation and in a karyotypically normal control boar (Table 4). No significant difference was observed between the (1;14) boar and the control (*p* ˃ 0.05), demonstrating the lack of interchromosomal effect (ICE) for this particular rearrangement (at least in the chromosomes studied).

**Table 4:** Analysis of interchromosomal effects: disomy, nullisomy, and diploidy rates for chromosomes 13, 18, X, and Y in sperm from control and t(1;14) boars

Chromosome number	Frequency of disomy % (n)		Frequency of nullisomy % (n)		Frequency of diploidy % (n)	
	Control	t(1;14)(q2.11;q1.2)	Control	t(1;14)(q2.11;q1.2)	Control	t(1;14)(q2.11;q1.2)
13	0.009 (2)	0	0.02 (4)	0.01 (1)	0.005 (1)	0
18	0.05 (11)	0.03 (3)	0.04 (9)	0.02 (2)	0.02 (5)	0
XY	0.04 (9)	0.03 (3)	0	0.01 (1)		
XX or YY	0.05 (11)	0.06 (6)				
Total no. of spermatozoa	20,226	10,127	20,226	10,127	20,226	10,127

*p* < 0.05 when compared with the value of the control for the same chromosome (chi-square test)

## Discussion

### Meiotic pairing and transcriptional abnormalities

As suspected, our analysis of meiosis I (prophase) spermatocytes from the t(1;14) boar revealed meiotic pairing abnormalities, characterized by the presence of γH2AX-modified histone on the quadrivalents or univalents and some association with the XY body. Such phenomena have been already described in humans (Ferguson et al. 2008; Leng et al. 2009; Sciurano et al. 2007, 2012). Sciurano et al. (2012) hypothesized that the movements of chromatin loops from autosomes which are actively transcribed, but not involved in the rearrangement, may randomly displace the inactive regions of the multivalent until they associate with the silenced chromatin domain of the XY body. Complementary RNA-DNA FISH experiments revealed a lack of transcriptional activity in the γH2AX-positive regions.

Microarray gene expression analysis revealed 2278 differential probes on t(1;14) of which 75 % corresponded to down-regulated genes. The two chromosomes with rearrangements (i.e., chromosomes 1 and 14) also had the largest number of down-regulated probes. Transcriptional activation was consistently detected on the X chromosome with 73 % of the differential probes being up-regulated and confirmed by real-time PCR for six genes distributed all along the X chromosome and corresponding to up-regulated probes. These data for gene expression concord with our observation that MSUC occurred in meiocytes from this boar and previous observations reported in mice (Homolka et al. 2007). Indeed, the autosome-autosome translocation studied by these latter authors was characterized by transcriptional down-regulation of the genes inside the unsynapsed region of the rearranged mouse autosome and an association between the quadrivalent and XY body, leading to X inactivation failure and incomplete silencing of the X chromosome genes in mid-late pachytene.

Despite the fact that we were working on frozen testis biopsies rather than purified germ cell populations, we were able to observe significant effects on gene expression that seemed to be related to MSUC (X reactivation and silencing of the rearranged chromosomes). This would explain the meiotic arrest of some spermatocytes and the observed oligospermia in this boar.

### Impact of the translocation on meiotic recombination

Eight human cases have been analyzed in the past using a comparable methodological approach involving immunolocalization of the MLH1 protein (Ferguson et al. 2008; Jiang et al. 2014; Leng et al. 2009; Oliver-Bonet et al. 2005; Pigozzi et al. 2005b; Sun et al. 2005; Wang et al. 2015). In four of these cases (t(Y;1), t(1;21), t(11;14), and t(5;7;9;13)), an effect of the rearrangement on the overall rate of recombination (increase or decrease) was observed (Leng et al. 2009; Pigozzi et al. 2005b; Sun et al. 2005; Wang et al. 2015). The other four cases (autosome-autosome reciprocal translocations) showed normal rates of recombination (Oliver-Bonet et al. 2005; Ferguson et al. 2008; Jiang et al. 2014) as did the only other reciprocal translocation (t(3;4)) studied in the pig species (Mary et al. 2016). In our study, a significant increase of the (average) overall recombination rate was observed in the t(1;14) boar, as compared with a pool of normal boars (controls). Different factors (genetic background, age, environment, etc.) might explain the observed differences. Furthermore, intraindividual and interindividual variability of the recombination rate has been demonstrated in humans and pigs with normal karyotypes (Hassold et al. 2004; Mary et al. 2014).

The specific analyses of the translocated chromosomes showed an increase in the MLH1 foci number and in the SC relative length for SSC1. This is consistent with a positive correlation between the number of MLH1 foci and the SC length observed in different species including humans (Pan et al. 2012), pigs (Mary et al. 2014), and mice with widely different rates of meiotic recombination (Baier et al. 2014).

Concerning the CO distribution on the paired SSC1 and SSC14 segments of the quadrivalents, significant differences were observed compared to their normal counterparts in control boars. Indeed, the recombination frequency was decreased in the vicinity of the breakpoint but appeared to be offset by an increase along the translocated chromosomes (Fig. 4). Similar results were obtained by Mary et al. 2016 for the t(3;4) translocation in pigs. This might indicate that as proposed by Mary et al. (2016), steric hindrance could occur in the breakpoint regions preventing the access of proteins involved in the recombination process. Abnormal meiotic pairing, not visible by immunolocalization (see Libuda et al. 2013) occurring in these regions, could also prevent the formation of CO around the breakpoint. Moreover, we can suspect that, as reported by Libuda et al. 2013 in *C. elegans*, partial depletion of the synaptonemal complex central region proteins at the breakpoint attenuates crossover interference, thereby increasing crossovers in the flanking regions. Finally, this decrease might also be offset in other autosomal pairs by an unknown factor.

### Genetically unbalanced gametes production

Five different segregation modes can occur in reciprocal translocation heterozygotes: alternate, adjacent I, adjacent II, 3:1, and 4:0 segregations, leading to the production of 18 possible types of gametes (Benet et al. 2005; Sybenga 1975). The relative frequencies of each segregation mode (as well as the relative frequencies of balanced and unbalanced segregation products) may vary from one translocation to the other, depending notably on the chromosomes involved, the location of the breakpoints, and the chiasma frequency (Rickards 1983). Sperm meiotic segregation studies performed in human carriers of balanced reciprocal translocations showed that 18.6–80.7 % of the spermatozoa were chromosomally unbalanced (Benet et al. 2005; Morel et al. 2004). Until now, sperm FISH meiotic segregation analyses for three reciprocal translocations have only been carried out in pigs (Kociucka et al. 2014; Massip et al. 2008; Pinton et al. 2004). One of these translocations (t(3;15)) was characterized by a small derivative chromosome (comparable in size to the der(14) chromosome observed in the present study) and a high proportion of 3:1 meiotic products (Pinton et al. 2004). Similar results, i.e., a high rate of genetically unbalanced gametes (73 %), mostly of the 3:1 type (44 %), were observed in the present case.

Meiotic pairing analysis of 284 spermatocytes revealed that 70 % formed a quadrivalent and 30 % of the cells exhibited a trivalent plus univalent configuration. CO distribution analysis (Fig. 5c) revealed a very low recombination frequency in the interstitial region (i.e., between the centromere and breakpoint) of chromosome 14 (red curve). Furthermore, no MLH1 signal was observed on the der(14) chromosome of 27.5 % of cells exhibiting a quadrivalent configuration. This suggests that der14 could segregate randomly to one of the gametes leading to a 3:1 segregation at the end of meiosis I. This is generally the case for the trivalent plus univalent configuration and would explain the relatively high rate of 3:1-like segregation meiotic products observed in our study.

Four different types of 3:1 segregations were observed (Table 4). Equivalent proportions of reciprocal segregation products would theoretically be expected for each category (e.g., similar proportions of “14” and “1^14^/1/14^1^” gametes). This was clearly not the case in our study (100 % of “14” gametes and 0 % of “1^14^/1/14^1^” gametes). Various authors (Estop et al. 1998; Rives et al. 2003; Van Assche et al. 1999) suggest that spermatocytes with fewer chromosomes would be better able to survive a 3:1 segregation. Such a phenomenon could partly explain our results.

**Fig. 7:**
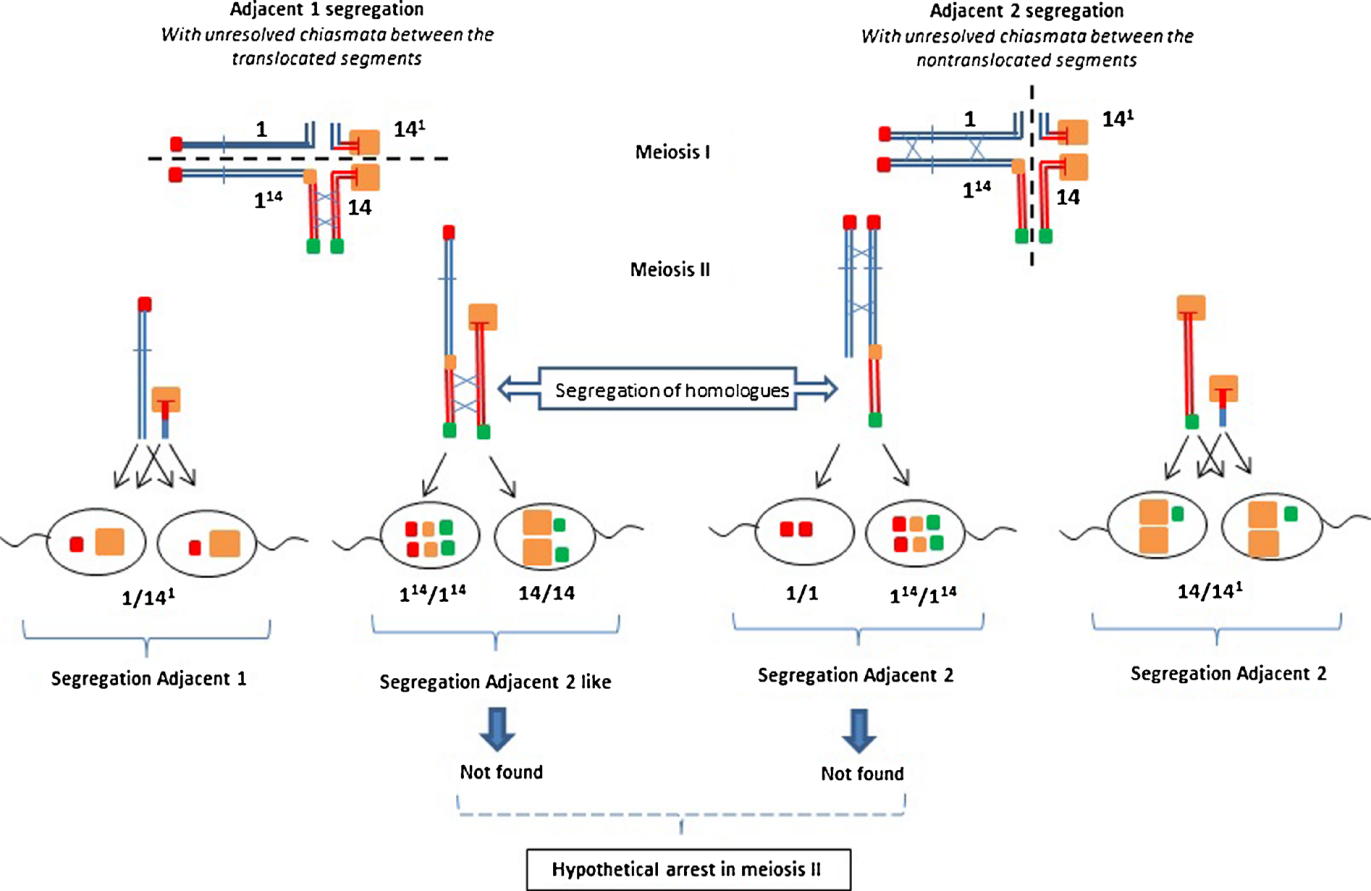
Illustration of sperm products from adjacent I segregation with unresolved chiasmata between translocated segments and from adjacent II segregation with unresolved chiasmata between non-translocated segments. Partial bivalents in meiosis II may then result in segregation of the homologous chromosomes instead of the chromatids which would produce different fluorescent sperm types or an arrest in meiosis II

As in earlier studies carried out in humans (Benet et al. 1989; Brandriff et al. 1986; Estop et al. 1992; Martin and Spriggs 1995; Shi and Martin 2001; Templado et al. 1988; Van Hummelen et al. 1997), we observed a deviation from the 1:1 ratio of reciprocal segregation products for the adjacent I and adjacent II segregations. These results might be explained by the presence of unresolved chiasmata at meiosis I (Nicklas 1977; Van Hummelen et al. 1997). Unresolved chiasmata would affect the translocated segments in adjacent I segregation and non-translocated segments in adjacent II segregation, resulting in partial bivalents in meiosis II, which could lead to the segregation of homologous chromosomes, or to meiosis II arrest (Mckim and Hawley 1995; Nicklas 1997). Thus, a higher frequency of products carrying the short translocated segment would be expected in adjacent I segregation and a higher frequency of products carrying the shorter non-translocated segment in adjacent II segregation. This is consistent with our observations (Fig. 7) that a large excess of sperm carried the short translocated segment (1/14^1^) among the adjacent I products and a smaller amount of sperm carried the shorter non-translocated segment (14^1^/14) among the adjacent II products. Moreover, the low frequencies (example 1^14^/14) or absence (example 1^14^/1^14^, 14/14, 14/14^1^) of some meiotic products can be expected to result from meiosis II arrest.

### Interchromosomal effect

As classically carried out in human studies (Anton et al. 2011; Douet-Guilbert et al. 2005; Machev et al. 2005; Piomboni et al. 2014; Shi and Martin 2001), ICE occurrence was investigated in chromosomes of different size and structure (SSC13 and SSC18, respectively, the largest and smallest acrocentrics, as well as the sex chromosomes). As in the study by Bonnet-Garnier et al. (2009) (investigation of ICE for two reciprocal translocations in the pig species), no ICE could be detected for any of the chromosomes tested. To our knowledge, the only publication reporting the occurrence of an ICE in the pig species is that of Pinton et al. (2004), which involved a boar mosaic for SSC18 trisomy. Unlike the situation in humans, very few cases have been investigated in the pig species until now, and complementary studies are needed to further document this point.

## Conclusion

The main goal of this study was to investigate the meiotic disturbances that resulted in the infertility of a young OAT boar carrying an autosome-autosome asymmetrical reciprocal translocation. Our results showed that the translocated autosomes exhibiting pairing defects sometimes associated with the XY body (even if the synapsis of these autosomal regions with sex chromosomal axes has not been proved by our approach) led to a reduction in the expression level of some autosomal genes, as well as to an up-regulation of some X-chromosome genes. We hypothesized that the main cause of the meiotic arrest might be the gene silencing of asynapsed autosomal regions and/or an up-regulation of some X chromosome genes. Further studies will be necessary to see if these phenomena (disturbance of autosomal and X gene expression) are dependent on the nature of the chromosomes involved in the translocation or on the lengths of the translocated chromosomal fragments.

## Electronic supplementary material

Below is the link to the electronic supplementary material.

Supplementary Table 1 Semen parameters of the control group and t(1;14) boar used for gene expression analysis (PDF 6 kb)

Supplementary Table 2 Primers used for qPCR analysis (PDF 6 kb)

Supplementary Fig. 1 Histopathological analysis of the testes sample (Hematoxylin and eosin stain) Histological analysis of the testes shows altered spermatogenesis with few spermatozoa in the lumen and diffuse hyperplasia of the interstitial cells. (x40 magnification) *Scale bar* is equal to 200 μm (JPG 41 kb)

Supplementary Fig. 2 Real-time PCR quantification of 6 genes located on the X chromosome and described as up-regulated by microarray analysis. A: Localization of the 6 genes on the X chromosome is shown by red dots. B: Fold change of expression in testis from t(1;14) boar compared to control boars. Results are the mean of three independent experiments performed with biological replicates. (*t-test*, ***p˂0.001; n.s. not significant) (JPG 63 kb)

## Abbreviations

AMCA: 1-Amino-4-methylcoumarin-3-acetic acid
BAC: Bacterial artificial chromosome
CO: Crossing-over
DE: Differentially expressed
FISH: Fluorescence in situ hybridization
FITC: Fluorescein isothiocyanate
HRP: Horse radish peroxidase
ICE: Interchromosomal effect
INRA: National Institute for Agricultural Research
lfc: Log-fold change
LH: Luteinizing hormone
MLH1: mutL homolog 1
MSCI: Meiotic sex chromosome inactivation
MSUC: Meiotic silencing of unsynapsed chromatin
OAT: Oligoasthenoteratospermia
PCA: Principal component analysis
PER V: Porcine endogenous retrovirus
SC: Synaptonemal complex
SCP1: Synaptonemal complex proteins 1
SCP3: Synaptonemal complex proteins 3
