# Supplementary material for: Meiotic pairing and gene expression disturbance in germ cells from an infertile boar with a balanced reciprocal autosome-autosome translocation
Source: Chromosome Res. 2016 Aug 2;24(4):511–27. doi: 10.1007/s10577-016-9533-9 (PMC5167775; doi:10.1007/s10577-016-9533-9)
Supplement: Supplementary file 1 — Semen parameters of the control group and t(1;14) boar used for gene expression analysis (PDF 6 kb) [file 10577_2016_9533_MOESM1_ESM.pdf]

	Name	Chromosomal status	Semen parameters	Concentration (10^6/mL)	Mobility (%)	Motility (1 to 5)
Control group	C1	38, XY	Normal	303	90	4
	C2	38, XY	Normal	660	90	4
	C3	38, XY	Normal	503	60	3,5
Translocated animal	T1-14	38, XY t(1;14)	Oligospermia<br>Asthenospermia<br>Teratospermia	19	38	1,5
