# Supplementary material for: Meiotic pairing and gene expression disturbance in germ cells from an infertile boar with a balanced reciprocal autosome-autosome translocation
Source: Chromosome Res. 2016 Aug 2;24(4):511–27. doi: 10.1007/s10577-016-9533-9 (PMC5167775; doi:10.1007/s10577-016-9533-9)
Supplement: Supplementary file 2 — Primers used for qPCR analysis (PDF 6 kb) [file 10577_2016_9533_MOESM2_ESM.pdf]

Gene		Sequence 5'-3'
RPL4	F	CAAGAGTAACTACAACCTTC
	R	GAACTCTACGATGAATCTTC
ARAF1	F	TCTCTGCCCTGATACTGCCT
	R	ATCCCTGCCACAAATCCCAG
LAMP2	F	CCAGGGTTTGTTGTTGTCGC
	R	CCTTACTCCAGCAGCCAGAG
RGN	F	GGGTGGCCTGTTACAATGGA
	R	GGTTGCTGCAAAAGACCCTG
SAT1	F	AGCGAAGAGGGATGGAGACT
	R	AACATGCAACAACGCCACTG
T5C22D3	F	CAGCTGGGACTTCACATCGT
	R	CTGAGCACATTACCCAGGCA
ZIC3	F	AACCGTGCCAAAGTCCTGTT
	R	TTCCCGGCACTTGTAGCAAT
