# Supplementary figures and images for: Meiotic pairing and gene expression disturbance in germ cells from an infertile boar with a balanced reciprocal autosome-autosome translocation
Source: Chromosome Res. 2016 Aug 2;24(4):511–27. doi: 10.1007/s10577-016-9533-9 (PMC5167775; doi:10.1007/s10577-016-9533-9)

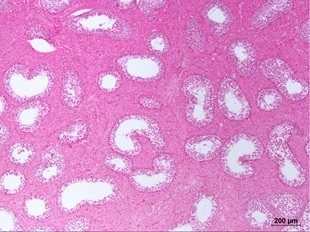

Supplement: Supplementary file 3 — Histopathological analysis of the testes sample (Hematoxylin and eosin stain) Histological analysis of the testes shows altered spermatogenesis with few spermatozoa in the lumen and diffuse hyperplasia of the interstitial cells. (x40 magnification) Scale bar is equal to 200 μm (JPG 41 kb) [file 10577_2016_9533_MOESM3_ESM.jpg]

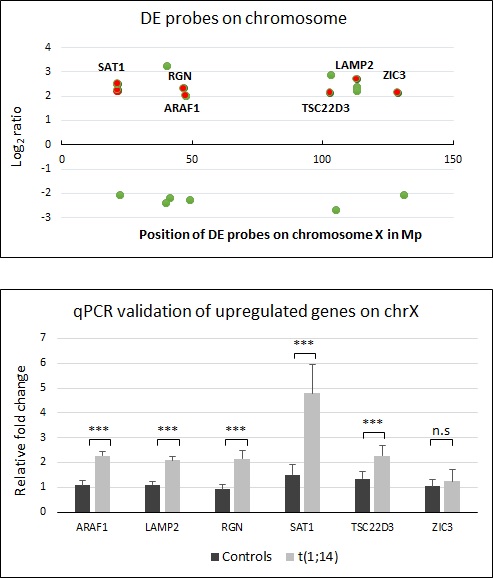

Supplement: Supplementary file 4 — Real-time PCR quantification of 6 genes located on the X chromosome and described as up-regulated by microarray analysis. A: Localization of the 6 genes on the X chromosome is shown by red dots. B: Fold change of expression in testis from t(1;14) boar compared to control boars. Results are the mean of three independent experiments performed with biological replicates. (t-test, ***p˂0.001; n.s. not significant) (JPG 63 kb) [file 10577_2016_9533_MOESM4_ESM.jpg]
